# Immune-stimulatory (TK/Flt3L) gene therapy opens the door to a promising new treatment strategy against brainstem gliomas

**DOI:** 10.18632/oncotarget.27834

**Published:** 2020-12-15

**Authors:** Syed M. Faisal, Flor M. Mendez, Fernando Nunez, Maria G. Castro, Pedro R. Lowenstein

**Affiliations:** ^1^Department of Neurosurgery, University of Michigan Medical School, Ann Arbor, MI 48109, USA; ^2^Department of Cell and Developmental Biology, University of Michigan Medical School, Ann Arbor, MI 48109, USA

**Keywords:** immunotherapy, gene therapy, thymidine kinase, Flt3L, DIPG

## Abstract

Diffuse intrinsic pontine glioma (DIPG) is a rare brainstem tumor which carries a dismal prognosis. To date. there are no effective treatments for DIPG. Transcriptomic studies have shown that DIPGs have a distinct profile compared to hemispheric high-grade pediatric gliomas. These specific genomic features coupled with the younger median age group suggest that DIPG is of developmental origin. There is a major unmet need for novel effective therapeutic approaches for DIPG. Clinical and preclinical studies have expanded our understanding of the molecular pathways in this deadly disease. We have developed a genetically engineered brainstem glioma model harboring the recurrent DIPG mutation, activin A receptor type I (ACVR1)-G328V (mACVR1) using the sleeping beauty transposon system. DIPG neurospheres isolated from the genetically engineered mouse model were implanted into the pons of immune-competent mice to assess the therapeutic efficacy and toxicity of immunostimulatory gene therapy using adenoviruses expressing thymidine kinase (TK) and fms-like tyrosine kinase 3 ligand (Flt3L). Immunostimulatory adenoviral-mediated delivery of TK/Flt3L in mice bearing brainstem gliomas resulted in antitumor immunity, recruitment of antitumor-specific T cells, and improved median survival by stimulating the host antitumor immune response. Therapeutic efficacy of the immunostimulatory gene therapy strategy will be tested in the clinical arena in a Phase I clinical trial. We also discuss immunotherapeutic interventions currently being implemented in DIPG patients and discuss the profound therapeutic implications of immunotherapy for this patient populations.

## INTRODUCTION

The World Health Organization (WHO) classification of CNS tumors has started to integrate molecular testing as part of its diagnostic criteria [1]. This led the WHO to classify a new neoplastic entity defined as midline gliomas (e.g., brainstem, thalamic, spinal cord) harboring H3K27M mutations and exhibiting diffuse growth, as H3K27M-mutant diffuse midline gliomas [1–4]. Amongst midline high grade gliomas, Diffuse Intrinsic Pontine Glioma (DIPG) is a highly aggressive and malignant pediatric brain tumor that develops in the brainstem. These tumors mainly arise in children, with peak incidence rates occurring between ages 6 and 9 years [5–7]. DIPGs account for half of childhood high grade gliomas (HGGs) and 85% of gliomas arising in the brainstem [5, 8]. Due to their location, surgical resection is not possible. Transcriptomic analyzes showed that DIPGs have a distinct molecular profile from other high-grade pediatric gliomas. The pathological assessment of DIPG identifies a diffuse pons tumor, sometimes infiltrating the medulla and midbrain [9]. To date, no definitive chemotherapeutic treatment appears to be successful in DIPG. Fractionated focal radiotherapy remains the only treatment capable of reducing tumor progression; however, this treatment is mostly palliative [8, 10, 11]. Additionally, radiation therapy elicits severe cognitive and functional impairments in children, due to damage to the developing brain. Consequently, DIPGs are the leading cause of pediatric brain tumor death [10]. The median survival is around 9 to 12 months, with 2-year survival rates around 10% and 5-year survival rates decreasing to less than 2% [5, 7, 8, 11, 12].

## PATHOPHYSIOLOGY AND GENETIC LESIONS

Intense research efforts over the past decade, have led to the discovery of unique mutations driving DIPG. The most frequent mutations affect the N-terminal tail of histone H3.3 and histone H3.1 and result in the change of a lysine to methionine at residue 27 [2–4]. It has been reported that the K27M mutation inhibits Enhancer of Zeste 2 (EZH2) histone methyltransferase activity causing a global hypomethylation at H3K27 displaying low levels of H3K27me^3^ [13]. DIPGs are also characterized by mutations in ACVR1^G328V^ (mutated in 24% of DIPG cases) [14–17]. ACVR1 encodes a type 1 BMP receptor and the six mutations reported result in constitutive BMP pathway activation [14–17]. Other mutations in DIPG include mutations in targeting tumor protein p53 (TP53), PIK3CA/PIK3R1, and PDGFRα amplifications [17, 18]. These unique set of mutations confer specific biological properties to the cancer cells themselves and they also impact the tumor microenvironment. This leads to the possibility of developing patient specific therapeutic opportunities and also challenges.

## PRECLINICAL ANIMAL MODELS OF DIPG

In order to develop and test novel therapeutic modalities for DIPG, it is imperative to have access to accurate preclinical mouse models, in which to test both efficacy and safety. Also, mouse models reflecting the genetic lesions and biology of DIPG are crucial for the development of targeted therapies to improve the prognosis of this devastating brainstem cancer. The first models of DIPG were xenograft models derived from tissue acquired at time of autopsy or biopsy material of DIPG patients [19]. Xenograft models are valuable because they have standardized growth rates, consistent times of death, and desired tumor localization, however, studies must be performed in immunocompromised mice which limits the ability to study the efficacy of immunotherapies. To overcome this limitation, a number of genetically engineered mouse models (GEMMs) of DIPG have been developed. Several GEMMs use a replication-competent avian sarcoma-leukosis virus long terminal repeat splice acceptor (RCAS) in order to achieve specific expression of oncogenes in cells expressing the avian cell surface receptor [19]. This model has been used to model the H3.3^K27M^ mutation by overexpressing PDGF-β, H3.3^K27M^, and loss of p53 in nestin progenitors in the brainstem [20]. In addition, models of mutated ACVR1 and H3.1^K27M^ and loss of p53 in nestin progentiors in the brainstem have also been generated using the RCAS/tva system [21]. Models using in utero electroporation to deliver PiggyBac DNA transposon plasmids expressing H3.3^K27M^ in neural progenitor cells have also been developed [22, 23]. Marigil et al. recently developed a guide-screw system based DIPG xenograft model, which allows the generation of tumors in a fast and reproducible fashion and allows to deliver the therapeutics via the same screw fixed system route [6, 24, 25]. Smith et al. recently performed a comprehensive histopathological and molecular analysis of 37 novel patient-derived orthotopic xenograft (PDOX) models developed from pediatric brain tumor patients [26], associated datasets can be accessed at (http://pbtp.stjude.cloud). We developed a DIPG model bearing mutated ACVR1^G328V^ or H3.1^K27M^, using the Sleeping Beauty transposon system to target neural stem cells in either the lateral ventricle or the fourth ventricle zone [27, 28]. Targeting the stem cells that line the fourth ventricle ensures the glioma will develop in the brain stem [27, 28]. From the genetically engineered model we generated neurospheres that enabled the development of an intracranial model of brainstem glioma through implantation of tumor neurospheres into the pons of adult C57BL/6 mice. This implantation model exhibits an intact immune system, it is highly invasive and displays 100% penetrance and short latency enabling it to be used for pre-clinical testing of targeted therapies and immune-mediated strategies for DIPG [27].

## IMMUNE-STIMULATORY GENE THERAPY

**Figure 1:**
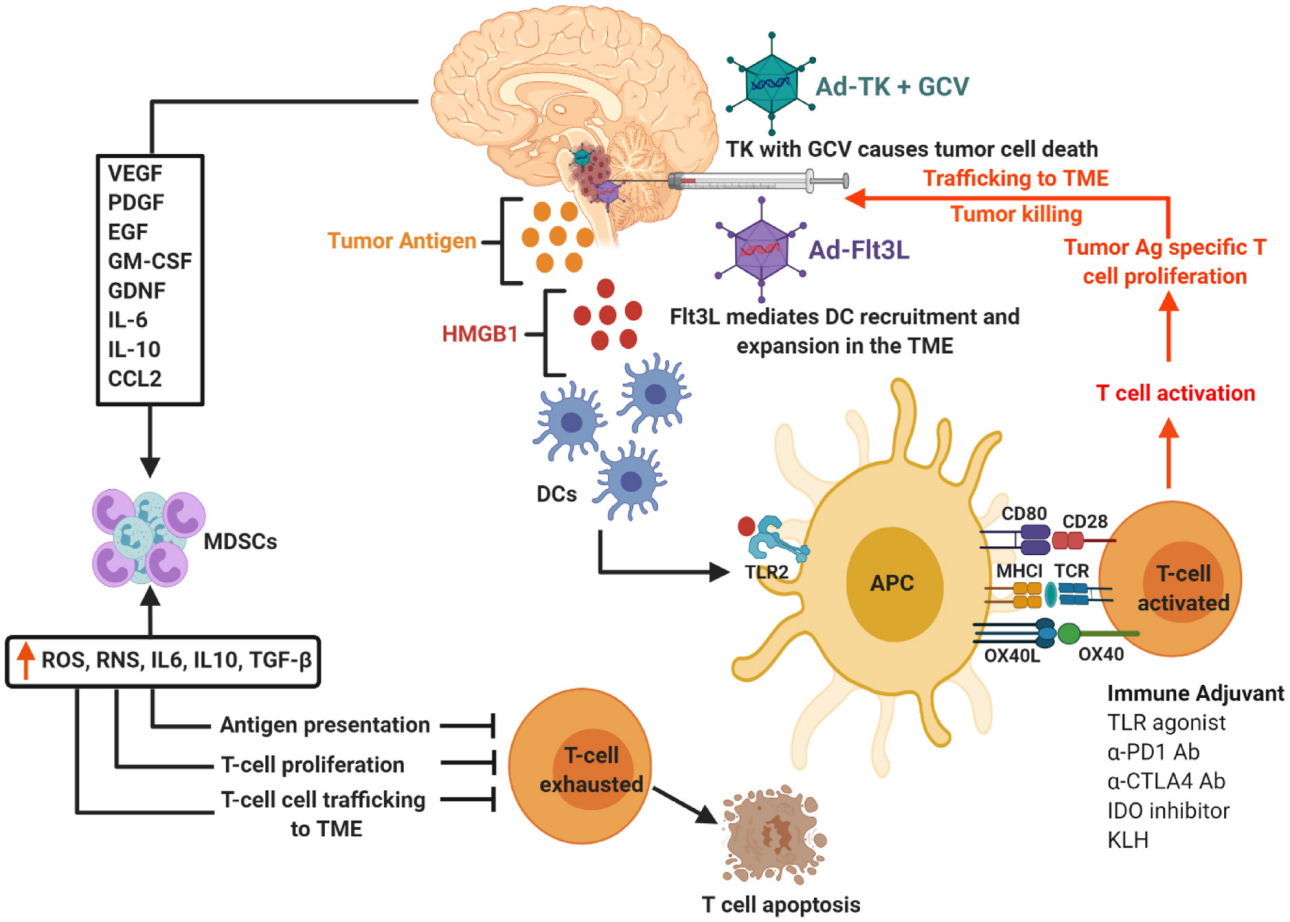
Schematics of TK/Flt3L based immunostimulatory gene therapy and underlying anti-DIPG immune mechanism. Tumor cells transfected with Ad-Flt3L express Flt3L protein enter systemic circulation. In the bone marrow (BM), Flt3L induces the expansion of dendritic cells (DCs), followed by their recruitment and accumulation in the tumor microenvironment (TME). Ganciclovir, which is a prodrug (GCV) is administered systemically. Tumor cells transfected with Ad-TK express TK protein capable of converting GCV to GCV-monophosphate (GCVp), which is further phosphorylated to GCV-diphosphate (GCVpp) by cellular kinase guanylate kinase and to the active antimetabolite GCV-triphosphate (GCVppp) by cellular nucleoside diphosphokinase. GCVppp, is incorporated into the replicating DNA of tumor cells, resulting in DNA replication termination and cell death. This also leads to the concomitant release of damage associated molecular patterns (DAMPs), i.e., HMBG1, Calreticulin, and ATP from dying tumor cells. Recruited DCs uptake the DIPG tumor Ag released from the dying cells. HMGB1 binds to TLR2/4, which facilitates the production of cytokines and tumor antigen cross-presentation. The DCs loaded with tumor antigens migrate to the cervical draining lymph node (dLN) where they present tumor antigens (Ag) to naive T cells, priming tumor specific anti-glioma effector T cells. Primed effector T cells enter the bloodstream from dLN and migrate towards the TME and kill residual tumor cells. Cytokines (VEGF, PDGF, LIF, GDNF, IL-6, IL-10, CCL2) released by glioma cells supporting differentiation and expansion of immune suppressive immature myeloid cells (MDSCs). To block effective anti-tumor immune responses, MDSCs are recruited to the tumor microenvironment and circulate back to lymphoid organs. The differentiation, maturation, activation, and proliferation of T cells are disrupted by these MDSCs, ultimately leading to T cell exhaustion and death.

Due to their location, DIPGs are not resectable and are highly invasive, taking into consideration this we exploited the potential of immunostimulatory gene therapy strategy to implement an immune-mediated therapeutic approach for DIPG, which has the potential to be translated into the clinical arena. This strategy involves the use of adenovirus (Ad) mediated delivery of herpes simplex virus type 1-thymidine kinase (TK) and Fms-like tyrosine kinase 3 ligand (Flt3L), which are injected into the tumor mass. Upon administration of the prodrug ganciclovir (GCV), proliferating tumor cells expressing TK will convert GCV to its phosphorylated active metabolite, which is then further phosphorylated by intracellular kinases, inducing termination of DNA replication leading to immunogenic cell death. Dying tumor cells release damage associated molecular patterns (DAMPs) molecules such as high-mobility group B1 protein (HMGB1), calreticulin, and ATP [29–31]. Flt3L mediates recruitment of dendritic cells (DC) followed by DC activation via Toll-like receptor 2 (TLR-2)-mediated signaling which is stimulated by HMGB1 released by dying tumor cells into the tumor microenvironment (TME). Activated DCs uptake tumor antigens, and transport them to the draining lymph nodes, where they activate T cells, priming a robust anti-tumor cytotoxic and memory T cell response. This in turn leads to tumor regression and long-term immune-mediated survival of brain tumor bearing animals [29, 30]. Figure 1 demonstrates the schematics of TK/Flt3L based immunostimulatory gene therapy and the underlying anti-DIPG immune mechanism. The study by Mendez et al. demonstrated that the combined gene therapy strategy targeting the host tumor immune response inhibits tumor progression and improves median survival of mACVR1 DIPG bearing mice [27]. This combined conditionally cytotoxic immunostimulatory gene therapy approach for newly diagnosed GBM patients, has recently completed its Phase I clinical trial accrual at University of Michigan Medical School (NCT01811992) [29, 30, 32, 33], no severe adverse events were observed in adult patients. Thus, we anticipate that the encouraging survival data and safety profile will enable the translation of this therapeutic approach to treat DIPG patients in the near future.

## ONGOING CLINICAL TRIALS

The ineffectiveness of the current standard of care for DIPGs has led to the development of many novel experimental therapies. Currently, immunotherapies stand out as potential treatments due to their minimal invasiveness and comprehensive tumor-eradicating capability [6, 7, 25, 27, 34]. The DIPG microenvironment contains low levels of antigen presenting cells (APCs) and adaptive immune cells, contributing to the tumor’s ability to grow undetected by the immune system [27, 34, 35]. However, unlike other HGGs, DIPGs do not appear to have a highly immunosuppressive or inflammatory microenvironment [35]. These factors make immunotherapies strong candidates for DIPG treatment. Oncolytic adenoviruses have emerged as promising immunotherapies capable of effectively treating DIPGs [6, 7, 25, 34]. One virus in particular, DNX-2401, is currently in Phase I trials (NCT03178032). Replication of this virus is dependent on a defective Rb pathway, a pathway commonly dysregulated in DIPG [6, 7]. This allows the virus to selectively target tumor cells. The high degree of specificity is essential given the sensitive anatomical location of these neoplasms. After infection, the virus replicates and eventually kills the infected cells [6, 7].

As mentioned, the only known effective treatment for DIPG is focal radiation. Although radiation therapy can improve survival and quality of life, ultimately it is not curative and it can have severe adverse effects. It has been shown that DNX-2401 inhibits DNA repair machinery of infected cells [25]. Therefore, DNX-2401 can potentially enhance therapeutic efficacy when combined with radiation therapy. The increased antitumoral effect of combination therapy has been supported using both *in vitro* and *in vivo* models [25]. Also, the radiation does not appear to affect replication of DNX-2401, so both therapies can be used concomitantly [25]. This makes the above therapy clinically relevant because has the potential to enhance the efficacy of the current standard of care.

The DIPG TME is relatively immunodeficient, lacking many components necessary to initiate a strong adaptive anti-DIPG immune response [36]. The reduced amount of immune cells and inflammatory signaling in the TME could be partially responsible for the low efficacy of radiation therapy. The combination of both DNX-2401 and radiation has been shown to also increase infiltration of lymphocytes and expression of proinflammatory cytokines in the TME [25]. This immunological shift could contribute to the increased survival observed *in vivo* when using the combination of DNX-2401 and radiation compared to each individually.

## CONCLUSIONS AND FUTURE PROSPECTS

The preclinical results by Mendez et al. are promising and indicate that it would be feasible to successfully test our TK/Flt3L-mediated gene therapy in a Phase I clinical trial for DIPG patients. The fact that the Phase I clinical trial for adult glioblastoma (GBM), recently completed at our Institution, using TK/Flt3L immune-stimulatory gene therapy, demonstrated it is a safe therapeutic approach (NCT01811992), provides a strong rationale for working towards its implementation in DIPG patients. The promising results of the immune-mediated gene therapy might be further enhanced by combining it with immune checkpoint inhibitors, opening new avenues worthy of investigation. We hypothesize that a combinatorial approach aiming to activate anti-DIPG immune response used together with immune checkpoint blockade would provide an effective therapeutic strategy against this devastating brainstem high grade glioma. It would therefore be important to test the impact on therapeutic efficacy and long-term survival of combining programmed death ligand 1 (PDL1) or CTLA-4 blockade with TK/Flt3L gene therapy in our preclinical model as a prelude to combinatorial phase I clinical trials. As the phase I clinical trials of TK/Flt3L gene therapy have already been completed and clinical-grade reagents for checkpoint blockade inhibitors are readily available, translating this combinatorial approach from bench to bedside should be feasible in the near future. In conclusion, the highly encouraging results are reported by Mendez et al. have opened an exciting opportunity for DIPG patients that will be tested in clinical trials in the very near future.
